# Spermatogonial Stem Cell Niche and Spermatogonial Stem Cell Transplantation in Zebrafish

**DOI:** 10.1371/journal.pone.0012808

**Published:** 2010-09-20

**Authors:** Rafael Henrique Nóbrega, Caaj Douwe Greebe, Henk van de Kant, Jan Bogerd, Luiz Renato de França, Rüdiger W. Schulz

**Affiliations:** 1 Division of Developmental Biology, Department of Biology, Faculty of Science, Utrecht University, Utrecht, The Netherlands; 2 Laboratory of Cellular Biology, Department of Morphology, Institute of Biological Sciences, Federal University of Minas Gerais, Belo Horizonte, Brazil; 3 Research Group Reproduction and Growth in Fish, Institute of Marine Research, Bergen, Norway; Brigham and Women's Hospital, United States of America

## Abstract

**Background:**

Spermatogonial stem cells (SSCs) are the foundation of spermatogenesis, and reside within a specific microenvironment in the testes called “niche” which regulates stem cell properties, such as, self-renewal, pluripotency, quiescence and their ability to differentiate.

**Methodology/Principal Findings:**

Here, we introduce zebrafish as a new model for the study of SSCs in vertebrates. Using 5′-bromo-2′-deoxyuridine (BrdU), we identified long term BrdU-retaining germ cells, type A undifferentiated spermatogonia as putative stem cells in zebrafish testes. Similar to rodents, these cells were preferentially located near the interstitium, suggesting that the SSC niche is related to interstitial elements and might be conserved across vertebrates. This localization was also confirmed by analyzing the topographical distribution of type A undifferentiated spermatogonia in normal, *vasa::egfp* and *fli::egfp* zebrafish testes. In the latter one, the topographical arrangement suggested that the vasculature is important for the SSC niche, perhaps as a supplier of nutrients, oxygen and/or signaling molecules. We also developed an SSC transplantation technique for both male and female recipients as an assay to evaluate the presence, biological activity, and plasticity of the SSC candidates in zebrafish.

**Conclusions/Significance:**

We demonstrated donor-derived spermato- and oogenesis in male and female recipients, respectively, indicating the stemness of type A undifferentiated spermatogonia and their plasticity when placed into an environment different from their original niche. Similar to other vertebrates, the transplantation efficiency was low. This might be attributed to the testicular microenvironment created after busulfan depletion in the recipients, which may have caused an imbalance between factors regulating self-renewal or differentiation of the transplanted SSCs.

## Introduction

Spermatogenesis is a cellular developmental process by which self-renewing spermatogonial stem cells (SSCs) differentiate into millions of sperm daily [1], [2]. To sustain this process continuously throughout the male reproductive life span, SSCs reside within a specific microenvironment in the testes called “niche” which regulates their properties, such as, self-renewal, pluripotency, quiescence and their ability to differentiate [3]–[5]. Despite of its crucial importance on SSC fate, the cellular and molecular composition of SSC niche remain unknown for several species of vertebrates. In rodents, the SSC niche has recently been identified within regions of the seminiferous tubules which are adjacent to the interstitial compartment [6], [7], preferentially along the branches of the interstitial blood vessels [8]. It has been hypothesized that the cellular and molecular environment near the interstitial compartment promotes SSC renewal, and when SSCs leave these areas, the associated changes in their environment promote SSC differentiation [5]. The proximity of SSC niche to the interstitium perhaps reflects the vascular supply of oxygen, nutrients, or hormones, such as follicle-stimulating hormone (FSH) or luteinizing hormone (LH) which influence Leydig and Sertoli cell functions on SSC self-renewal and also on SSC retention and homing in the niche [5], [9]–[11]. For example, FSH induces the secretion of GDNF (glial cell-line derived neurotrophic factor), an extrinsic stimulator of SSC self-renewal, produced by Sertoli cells [3], [12].

Currently, the only means to study SSCs and their niche is by exploiting the stem cells' functional properties, such as slow-cycling and quiescent nature through the label-retaining cell (LRC) approach [13], or by studying SSC functionality and plasticity by transplantation assays. In this context, transplantation techniques developed by Brinster and collaborators [14], [15] has enabled tremendous progress in the phenotypic and functional investigations of SSCs. Nowadays, SSC transplantation approaches have been developed for a number of species including also teleost fish [16], [17]. These data have broad implications for understanding the regulation of spermatogenesis, stem cell biology, etiology of male infertility [18]–[22], and also for advancing biotechnologies such as conservation of valuable genetic stocks, preservation of endangered species, and also as new option for transgenesis [16], [17], [19].

**Figure 1:**
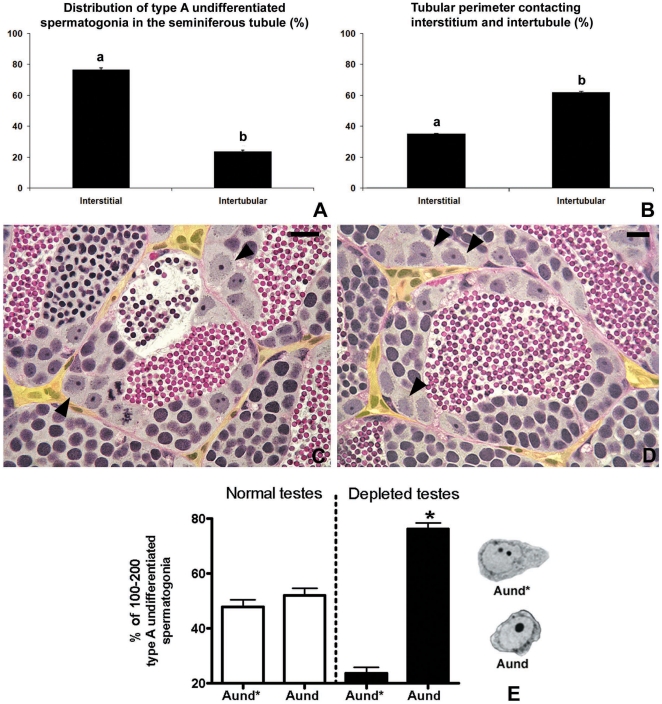
Topographical distribution of type A undifferentiated spermatogonia in zebrafish testes. **A.** Quantification of type A undifferentiated spermatogonia as located near the interstitium or in the intertubular area. Note that ∼76% of type A undifferentiated spermatogonia are preferentially located near the interstitium. **B.** Tubular perimeter of the regions contacting the interstitium or intertubular areas. **A,B.** Bars represent the mean ± SE which are expressed as percentage. Different letters mean significant differences among the groups. **C,D.** Histological sections of the zebrafish seminiferous tubules. Note that most of type A undifferentiated spermatogonia (arrowheads) are distributed near the (yellow) interstitium. Staining: PAS (Periodic Acid Schiff)/Ferric Hematoxylin/Metanil Yellow. Scale bar  = 10 µm. **E.** Relative number of type A_und*_ and A_und_ in normal (n = 7) and busulfan-depleted testes (n = 10). Bars represent the percentage mean ± SE of 100–200 type A spermatogonia, significant differences among the groups (p<0.05) are indicated by an asterisk.

**Figure 2:**
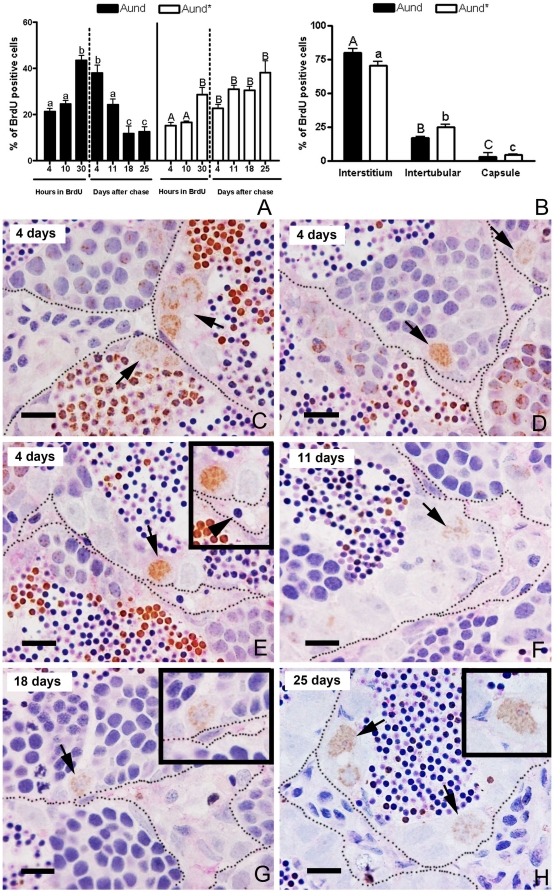
Label retaining cell (LRC) approach in zebrafish testes. **A.** BrdU labeling index of type A_und*_ and A_und_ during BrdU exposure (4, 10, and 30 h) and after 4, 11, 18 and 25 days of chase. Bars represent the percentage mean ± SE (n = 5); different letters denote significant differences (p<0.05) in time. **B.** Distribution of BrdU-positive A_und*_ and A_und_ spermatogonia after 18 and 25 days of chase. Bars represent the mean ± SE which are expressed as percentage, and different letters indicate significant differences (p<0.05) among the groups. **C**–**H.** BrdU immunodetection with PAS staining after 4 (**C**–**E**), 11 (**F**), 18 (**G**), and 25 (**H**) days of chase. Note that BrdU immunostaining is diluted as a consequence of the progression of spermatogenesis. Only the slow-cycling cells (stem cells candidates) are able to retain the BrdU label for long periods of time. LRCs are indicated by arrows. Some of LRC are near to blood vessels (**arrowhead**) (**E**). Insets are high magnification of the LRCs (type A undifferentiated spermatogonia). The interstitium is delimited by spotted lines. Scale bars  = 10 µm.

**Figure 3:**
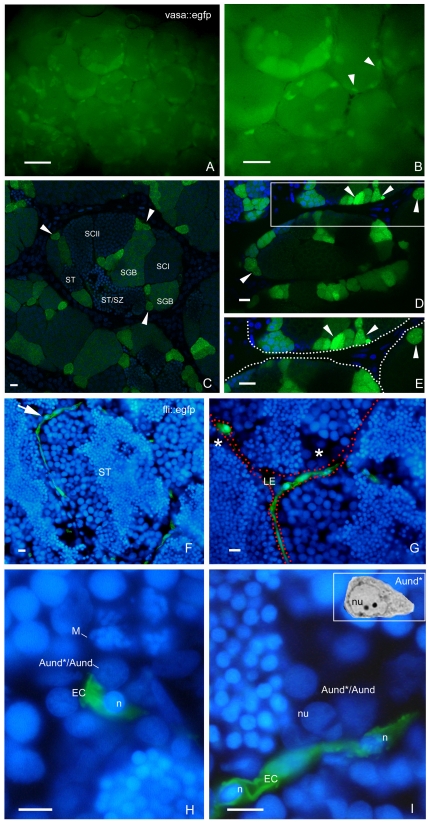
SSC niche in zebrafish testes. Whole-mount of *vasa::egfp* testes analyzed under fluorescence (**A,B**) and CLSM (**C**–**E**). **A,B.** Most of the brightest and small spots (type A undifferentiated spermatogonia) (arrowheads) are adjacent the triangle/lozenge dark areas (interstitium). Scale bars  = 0.1 mm and 0.5 mm, respectively. **C**–**E.** Vasa is highly expressed in type A undifferentiated spermatogonia, and is gradually decreased during the spermatogenesis. A_und*/_A_und_ (arrowheads), type B spermatogonia (SGB), primary spermatocytes (SCI), secondary spermatocytes (SCII), spermatids (ST), spermatids and spermatozoa (ST/SZ). Note that most of A_und*/_A_und_ (arrowheads) are situated near the interstitium (**D**, dark areas; **E**, delimited by spotted lines). **E** is a high magnification of the square in **D**. Scale bars  = 10 µm. **F**–**I:** Cryosections of *fli::egfp* testes stained with DAPI (nuclear staining) and analyzed under fluorescence microscopy. The arrow in **F** shows a blood vessel (green) surrounding the circumference of a seminiferous tubule (ST). Interstitium (delimited with red dotted lines), Leydig cells (LE) and group of type A spermatogonia (asterisks) are shown in **G**. **H,I.** A_und*/_A_und_ are near the endothelial cell (EC) nuclei (n). Note a metaphase figure (**M**) in **H**. Nucleolus (nu) of type A undifferentiated spermatogonia is shown in **I**. Compare the similar morphology of A_und*_ (inset) with the cells found near the endothelial cell. Scale bars  = 10 µm.

In anamniote vertebrates (fishes and amphibians), we find the cystic type of spermatogenesis [2]. There are two main differences compared to higher vertebrates. First, within the spermatogenic tubules, cytoplasmic extensions of Sertoli cells form cysts that envelope a single, clonally and hence synchronously developing group of germ cells deriving from a single spermatogonium. Second, the cyst-forming Sertoli cells retain their capacity to proliferate also in adult fish [23], [24]. Hence, the basic functional unit of the spermatogenic epithelium in fish is a spermatogenic cyst formed by a dynamic group of Sertoli cells surrounding and nursing one synchronously developing germ cell clone. Different clones being in different stages of development generate the typical histological picture of fish testes, where the tubular compartment contains cysts of different sizes with groups of germ cells in different stages of spermatogenesis (see Figures 1, 2 and 3). Detailed histological studies in zebrafish (*Danio rerio*) have described two subtypes of type A undifferentiated spermatogonia in the testes, designated as A_und*_ and A_und_ [23]. It is not known if these two subtypes are separated by mitosis, or represent different stages of the same cell cycle, and moreover, there is no information on the spermatogonial stem cell niche in the zebrafish testis, or on the stemness of A_und*_ and A_und_.

In the current study, we identified the putative SSCs and their niche by identifying the LRCs in zebrafish testes and using a transgenic zebrafish expressing enhanced green fluorescent protein under the control of the germ cell specific *vasa* promoter (*vasa::egfp*) [25]. Furthermore, we evaluated the spatial relationship between blood vessels and the SSC niche, using testes expressing enhanced green fluorescent protein under the control of the endothelial cell specific *fli* promoter (*fli::egfp*) [26]. To confirm the biological activity (re-establishment of function and plasticity) of the potential SSCs, we developed a transplantation assay in zebrafish. Finally, we studied the recipient's testicular microenvironment prior to transplantation as it might influence the behavior of transplanted SSCs.

## Materials and Methods

### Animals

Sexually mature zebrafish males and females, and sexually mature transgenic zebrafish males expressing enhanced green fluorescent protein under the control of the germ cell-specific *vasa* promoter (*vasa::egfp*) [25] or the endothelial cell-specific *fli* promoter (*fli::egfp*) [26] were used. Animal housing and experimentation were consistent with Dutch and Brazilian national regulations and were approved by the Utrecht University and Federal University of Minas Gerais animal use and care committees, respectively.

### Topographical distribution of type A undifferentiated spermatogonia in zebrafish seminiferous tubules

Testes from males (n = 5) were fixed in 4% buffered glutaraldehyde at 4°C overnight, dehydrated, and embedded in Technovit 7100 (Heraeus Kulzer, Wehrheim, Germany, http://www.kulzer-technik.de), sectioned and stained according to conventional histological procedures [23], [27]. The topographical distribution of A spermatogonia was recorded by examining if type A undifferentiated spermatogonia (A_und*_ or A_und_) were adjacent to the interstitial compartment, or contacted one or more tubules (intertubule). The position of 500 type A undifferentiated spermatogonia was counted per animal and expressed as percentage of the total number evaluated; to determine if the distribution of these cells follows a random pattern, the tubular perimeters adjacent to the interstitium, or intertubule were measured using Image J software (National Institutes of Health, Bethesda, Maryland, USA, http://rsbweb.nih.gov/ij), and the values were expressed as percentage of the total tubular perimeter (n = 50 tubules/animal).

### Identification and quantification of label retaining cells (LRCs) in zebrafish testes

To estimate the cell cycle duration of type A undifferentiated spermatogonia (A_und*_ and A_und_), 10 males were exposed to BrdU (Sigma-Aldrich, St. Louis, MO, USA, http://www.sigmaaldrich.com) dissolved in water (4 mg/ml) for approximately 4 and 10 h. To identify the LRC population, 25 males were pulsed with BrdU dissolved in water (4 mg/ml) for 10 h/day during 3 consecutive days. Animals (n = 5) were sacrificed immediately after the third BrdU pulse, and after 4, 11, 18 and 25 days of chase. Testes were fixed, embedded, and sectioned as described above. BrdU incorporation was detected by immunohistochemistry as described previously [28]. The labeling index of both types of A undifferentiated spermatogonia (A_und*_ or A_und_) was determined by counting the total number of labeled cells out of 300 cells (labeled and non-labeled) during the different periods after chase. After 18 and 25 days of chase, the spatial distribution of labeled A_und*_ or A_und_ (LRCs) was determined by counting the number of labeled cells situated near the interstitium, or in the intertubule, or near the testicular capsule. The values were expressed as percentage of positive-BrdU cells in the mentioned regions.

### Whole-mount analysis of *vasa::egfp* testes under confocal laser scanning microscopy (CLSM) and examination of *fli::egfp* testes

Testes from transgenic *vasa::egfp* [25] or *fli::egfp* [26] zebrafish were fixed in 2% buffered paraformaldehyde for 2 h. For whole-mount examination, *vasa::egfp* testes were permeabilized with 0.2% PBT (0.2% Triton X-100 in PBS) for 10 min, and subsequently stained in DAPI (Invitrogen Molecular Probes, Carlsbad, CA, USA, www.invitrogen.com) for 5 min, followed by two rinses in PBS for 10 min. *vasa::egfp* testes (n = 5) were analyzed by CLSM 510 Meta (Zeiss, Jena, Germany, www.zeiss.de/lsm) using 358 nm and 488 nm as excitation wavelengths for DAPI and GFP, respectively. *fli::egfp* testes were frozen in Tissue-Tek (Sakura Finetek Europe B.V., Leiden, Netherlands, http://www.sakura.eu) and cryosectioned at 10 µm, permeabilized in 0.2% PBT for 10 min, stained with DAPI (Invitrogen) for 5 min, and mounted with a coverslip using an anti-fading Vectashield mounting medium (Vector Laboratories, Burlingame, CA, USA, http://www.vectorlabs.com). Sections were examined under fluorescence microscopy using filters for DAPI and FITC visualization.

### Depletion of endogenous spermatogenesis in zebrafish male recipients for SSC transplantation

To deplete endogenous spermatogenesis, we first examined the effect of different temperatures. Overall, the speed of spermatogenesis in teleost fish is significantly influenced by temperature [29]. Hence, males were kept in water at 20°C (n = 15), 27°C (n = 12), 30°C (n = 7), or 35°C (n = 12) for at least one week. Then, animals received one single intraperitoneal injection of ^3^H-thymidine (Amersham/GE Healthcare, Piscataway, NJ, USA, http://www.gehealthcare.com) (2 µCi/g/BW), and were sacrificed at 2 h, 12 h, 1, 2, 3, 4, 5 and 6 days after thymidine injection. Testes were weighed for calculating the gonadosomatic index (GSI  =  testes weight/body weight ×100), fixed, embedded and sectioned as above, and prepared for autoradiographic analysis to estimate the duration of meiosis and spermiogenesis as described previously [16], [29]. Based on these results (Figure S1), 35°C was chosen as optimal temperature to deplete endogenous zebrafish spermatogenesis. Then, 60 zebrafish males were kept at 35°C for one week, and received a single intraperitoneal dose of 30 or 40 mg/Kg/BW of busulfan (Sigma). Testes from males (n = 6) were sampled 2, 4, 6, 10 and 12 days after the injection, weighed, fixed, embedded, sectioned and stained as above. As control group, males (n = 30) received a single intraperitoneal injection of dimethyl sulfoxide and sampled at the same reported periods. To evaluate the optimal dose and the best window of depletion, frequency of spermatogenic cysts, germ cell apoptosis, and Sertoli cell only phenotype were determined for each sampled period. Results were expressed as percentage of the total number of counted structures. To address the composition of type A undifferentiated spermatogonia (A_und*_ or A_und_) in busulfan-depleted testes (n = 10), the percentage of both subtypes was determined by counting the number of A_und*_ and A_und_ out of 100–200 type A undifferentiated spermatogonia in each male. As control, the percentage of both subtypes was also determined in normal testes (n = 7).

### Preparation of zebrafish female recipients for SSC transplantation

Ovoposition was induced according to usual procedures (http://zfin.org). To evaluate if females after ovoposition may be suitable recipients for SSC transplantation, ovaries (n = 5) were fixed, embedded, and sectioned as above, and stained with PAS to determine the number of postovulatory follicles (POFs)/mm^2^ of tissue. POFs consisting for a great part of granulosa cells remaining after ovulation were considered as a space potentially available for transplanted SSCs. The number of POFs/mm^2^ from preovulatory females was also evaluated as a control.

### Donor cell preparation and SSC transplantation into male and female zebrafish recipients

Testes from males (n = 10) were digested with 0.2% collagenase and 0.12% dispase [30]. The obtained cell suspension was immediately submitted to FACS (Fluorescence Activated Cell Sorting) using an inFlux cell sorter (BD Bioscience, San Jose, CA, USA, www.bdbiosciences.com). *Vasa* is highly expressed in type A undifferentiated spermatogonia, but expression decreases during meiosis and spermiogenesis [23]. Since type A undifferentiated spermatogonia are the largest germ cell type in zebrafish testes (∼10 µm nuclear diameter [23]), FACS settings were adjusted to sort a cell population displaying large size and high intensity of fluorescence, which should enrich type A undifferentiated spermatogonia. To validate the enrichment, the unsorted and sorted cell fractions obtained by FACS was analyzed under fluorescence microscopy, or fixed, embedded, sectioned, and stained as above to determine the percentage of germ cell type. For SSC transplantation, the FACS-enriched cell fraction was suspended in L-15 medium (Sigma), 5% trypan blue and 10% calf serum. SSCs were transplanted into testes or ovaries through the genital pores, using a glass capillary needle coupled to a peristaltic pump (Figure S2). To optimize this procedure, zebrafish male and female genital pores were analyzed morphologically (diameter and angle) to adjust the settings for the glass capillary needle (Figure S2). The transplantation route was standardized and tested by injecting trypan blue (Figure S2).

### SSC transplantation analysis

Recipients were sacrificed 2 and 3 weeks (males) or 3 and 4 weeks (females) after transplantation. The gonads were fixed in 2% buffered paraformaldehyde for 2 h, permeabilized, and stained with DAPI before analysis by CLSM, as described above. As positive control, Vasa protein expression was examined by GFP immunodection in *vasa::egfp* gonad sections or by Vasa immunocytochemistry in wild-type males [31]. The PCR detection of *gfp* DNA from donor-derived germ cells in male and female recipients was carried out as described previously [32].

### 11-Ketotestosterone (11-KT) plasma levels in busulfan-depleted male zebrafish and 11-KT release by depleted testes *in vitro*

Males (n = 19) were sampled 10 days after busulfan (40 mg/kg) treatment. A blood sample was collected for quantification of 11-ketotestosterone (11-KT) plasma levels, as described previously [33]. As controls, 11-KT plasma levels were also quantified in zebrafish kept at 27°C (n = 7), or at 35°C (n = 11). In other experiments, carried out at the same time, but published separately [33], 11-KT plasma levels were measured 2 h after a single injection of recombinant zebrafish Fsh or hCG. Results are expressed as ng 11-KT/ml of plasma. To evaluate testicular 11-KT release in tissue culture, testes were collected from adult zebrafish kept at 27°C (control) (n = 7), or from busulfan-treated zebrafish (n = 7). The two testes of a given fish were incubated in parallel, such that one of them (randomly chosen left or right) served as control (basal) for the contralateral one, which was incubated in the presence of 1 µM of the adenylate cyclase activator forskolin [33]. After incubation the medium was processed for the quantification of 11-KT [34]. Results were expressed as ng 11-KT/mg of tissue.

### Gene expression in spermatogenesis-depleted testes in busulfan-treated zebrafish

Testes from males kept at 27°C (control) (n = 7), or at 35°C (n = 5), or treated with busulfan (n = 12) were snap frozen in liquid nitrogen and stored at −80°C until RNA extraction. Total RNA was extracted from testes using the RNAqueous®-Micro Kit (Ambion, Austin, TX, USA, http://www.ambion.com). Further processing to determine the threshold cycle (Cq) values of the reference endogenous control gene *elongation factor 1-alpha* (*ef1α*) and *β-actin1*, as well as of *insulin-like 3 (insl3)* [35], *steroidogenic acute regulatory protein* (*star*), and *cytochrome P450, family 17, subfamily A, polypeptide 1* (*cyp17a1*), *androgen receptor* (*ar*), *anti-Müllerian hormone* (*amh*), *gonadal soma-derived growth factor (gsdf), insulin growth factor 1a (igf1a)* and *1b (igf1b)* [36], and germ cell genes *piwil1* (spermatogonia), and *synaptonemal complex protein 3 (sycp3l)* (spermatocytes) by qPCR analysis was performed as reported [33], [37], [38]. No significant differences (P>0.05) were found among the mean *β-actin1* and *ef1α* Cq values in the different groups (Figure S3) thus validating *β-actin1* and *ef1α* as suitable references for the current experiments. Then, relative mRNA levels of the selected genes were normalized to *β-actin1* and *ef1α*, and expressed as fold of relative control (27°C) mRNA levels. Nomenclature of zebrafish proteins, mRNAs and genes are according to ZFIN (http://zfin.org) rules.

### Statistical analysis

Significant differences between two groups were identified using Student test (paired and unpaired) (P<0.05). Comparisons of more than two groups were performed with one-way ANOVA followed by Student-Newman-Keuls test (P<0.05). Graph Pad Prism 4.0 (Graph Pad Software, Inc., San Diego, CA, USA, http://www.graphpad.com) was used for all statistical analysis.

## Results

### Characterization of SSC candidates and their niche in zebrafish testes

Analyzing the topographical distribution of type A spermatogonia showed that 75% of these cells are situated adjacent to the interstitial compartment (Figure 1A,C,D) although this compartment represents only 1/3^rd^ of the total perimeter of the spermatogenic tubules (Figure 1B). Quantification of the mitotic index of these cells was achieved by determining the BrdU labeling index during three successive 10 h long exposures to BrdU during three consecutive days. The results suggested that both subtypes of type A undifferentiated spermatogonia (A_und*_ and A_und_) have a long cell cycle lasting at least 10 h but less than 30 h (Figure 2A). To confirm the existence of slow-cycling cells (stem cell candidates), testes were examined for BrdU-positive type A_und*_ and A_und_ spermatogonia at different periods (up to 25 days) after the final BrdU exposure; putative stem cells are considered to be part of the LRC population. During this chase period, two distinct patterns of BrdU staining were found among the two subtypes of type A spermatogonia (Figure 2A). A_und_ rapidly lost the label until the 18^th^ day of chase, when the labeling index stabilized at ∼10% for this cell type (Figure 2A). On the other hand, the percentage of BrdU-positive type A_und*_ spermatogonia varied in a statistically not significant manner around 30% during the complete chase period (Figure 2A). Apart from some somatic elements, type A undifferentiated spermatogonia (A_und*_ and A_und_) were the only BrdU retaining germ cells after 18 and 25 days of chase. Intriguingly, most of the labeled spermatogonia (71% of A_und*_ and 80% of A_und_) were situated adjacent to the interstitium (Figure 2B,C–H). A similar pattern was found in *vasa::egfp* testes examined under CLSM where the strongest expression of *vasa* was observed adjacent to the interstitium (Figure 3 and Video S1). To further study the spatial relation between the vasculature and undifferentiated spermatogonia, testes of transgenic zebrafish expressing GFP in endothelial cells (*fli::egfp*) were analyzed. Capillaries surround the seminiferous tubules (Figure 3F), and type A undifferentiated spermatogonia were often found in close association with endothelial cells (Figures 2E-inset; 3G–I). A combination of the above results is illustrated schematically in Figure S4, showing a hypothetical spermatogonial stem cell niche in zebrafish.

### Preparation of male and female recipients for SSC transplantation

**Figure 4:**
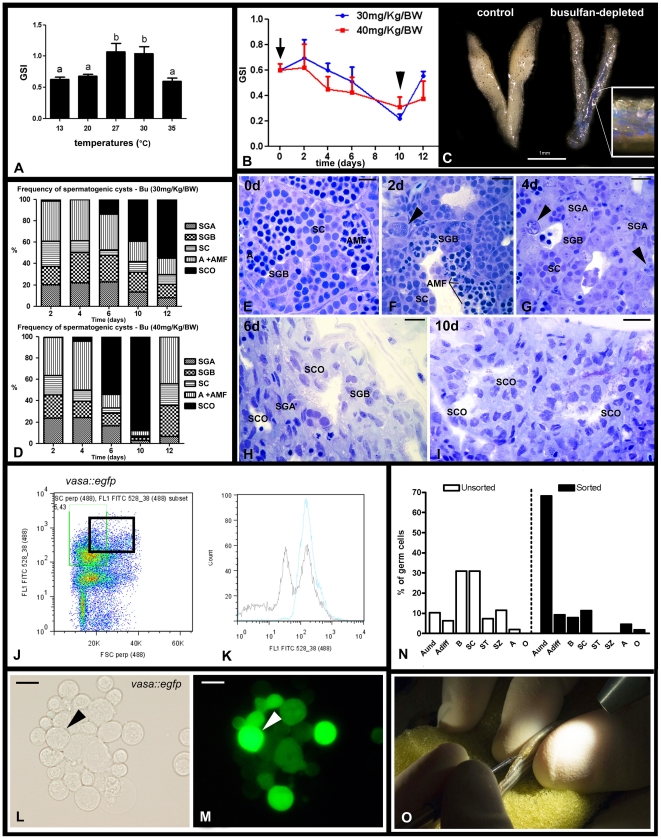
Depletion of endogenous spermatogenesis in male recipients for SSC transplantation. **A.** Effects of different temperatures on zebrafish GSI (gonadosomatic index). Bars represent mean ± SE (n = 15, 20°C), (n = 12, 27°C), (n = 7, 30°C), and (n = 12, 35°C). Different letters indicate significant differences (p<0.05) among groups. **B.** Effects of two single doses of busulfan (30 or 40 mg/Kg) on zebrafish GSI. The arrow indicates the day of injection (day 0), the arrowhead shows that the lowest GSI is observed 10 days after injection. Dots represent mean±SE (n = 6). **C.** Testes from control (27°C, left) and busulfan-depleted animals. Inset shows a high magnification of the depleted testis after in vivo injection via the urogenital pore of a solution containing trypan blue. **D.** Frequency of spermatogenic cysts after a single dose of 30 or 40 mg/Kg busulfan. Type A spermatogonia (SGA), type B spermatogonia (SGB), spermatocytes (SC), apoptosis and abnormal metaphase I figures (A+AMF), Sertoli cell only (SCO). Bars represent means expressed as percentage. **E**–**I.** Histological sections of testes collected at 0 (**E**), 2 (**F**), 4 (**G**), 6 (**H**), and 10 (**I**) days after a single injection of 40 mg/Kg busulfan at 35°C. Note that busulfan induced spermatogonial apoptosis (arrowheads) after 4 days of injection. Scale bars  = 10 µm. **J.** Testicular cell suspensions were obtained from *vasa::egfp* testes and subjected to FACS. Dot plot shows the total testicular suspension from which a population of large cells (forward scatter, FSC; abscissa), showing an intense fluorescence (FL1 FITC; ordinate) was sorted (black square). **K.** Histogram shows an enrichment of the sorted cells after FACS. Blue line (sorted cells), black line (total testicular cell suspension). FL1 FITC (x axis) means intensity of fluorescence, and counts (y axis), the number of events. **L,M.** Fraction of sorted cells under normal light (**L**) and under fluorescence (**M**). Arrow indicates a large cell carrying high fluorescence. Scale bars  = 10 µm. **N.** Histogram showing the percentage of germ cells in the unsorted and sorted fractions. Despite of the contamination with other germ cell types (cell clumping before FACS), there is an enrichment of type A undifferentiated spermatogonia (A_und*/_A_und_) population, which might contain SSC candidates. Undifferentiated type A spermatogonia (A_und*_ and A_und_), differentiating type A spermatogonia (A_diff_), type B spermatogonia, spermatocytes (SC), spermatids (ST), spermatozoa (SZ), apoptosis (A) and others (O). Bars represent means expressed as percentage. **O.** Germ cell transplantation into zebrafish genital pore using a glass capillary needle.

Zebrafish were exposed to different temperatures to optimize the treatment with busulfan (Figure 4A, Figure S1). Higher temperatures accelerated spermatogenesis (Figure S1), whereas at 35°C, spermatogenesis did not progress beyond metaphase I and showed abnormalities, such as massive apoptosis in particular amongst spermatocytes, absence of sperm, and a significant decrease in the GSI (Figure 4A,E; Figure S1). Both doses of busulfan (30 or 40 mg/Kg/BW) tested at 35°C induced further and a more general germ cell apoptosis (Figure 4D–H) and a progressive GSI decrease, which reached its lowest value 10 days after injection (Figure 4B,C). However, only the higher dose suppressed efficiently endogenous spermatogenesis, resulting in 88% of the spermatogenic tubules showing a Sertoli cell only appearance, i.e. all germ cells were missing (Figure 4D,I). Analyzing the composition of type A spermatogonia in the 12% of tubules where some germ cells remained and comparing this to normal testes demonstrated that the ratio between A_und*_ and A_und_ shifted from 1∶1 to 1∶3 in busulfan-depleted testes (Figure 1E). The preponderance of A_und_ might be associated with the observation that 12 days after injection, most tubules showed the first type B spermatogonia and/or spermatocytes again, i.e. showed a fast recovery of endogenous spermatogenesis after treatment with 40 mg/Kg/BW of busulfan at 35°C (Figure 4D).

To prepare female zebrafish recipients for SSC transplantation, ovoposition was induced; a pilot study indicated a high mortality among females when applying the combination of high temperature and busulfan. The increased number of POFs showed that ovoposition created spaces and “free” somatic cells (follicle cells from POFs), potentially suitable for receiving injected germ cells, and to support transplanted SSCs development, respectively (Figure S5).

### Donor cell isolation and male and female transplantation

Using *vasa::egfp* transgenic zebrafish as donors, a testicular cell suspension was obtained and subsequently submitted to FACS, in order to enrich transplantable SSC (Figure 4J,K) by sorting for big cells carrying high fluorescence (Figure 4L,M). Indeed, after sorting, we observed an enrichment of type A undifferentiated spermatogonia (Figure 4K–N). The sorted cells (8.10^3^−4.10^4^ cells/µl) were injected through the genital pore (Figure 4O, Figure S2). Since females have a prominent belly, direct injections into ovaries (via lateral body wall) were also successfully performed (Figure S2). After transplantation, zebrafish recipient males and females were placed in water of 27°C.

### Male and female transplantation analysis

**Figure 5:**
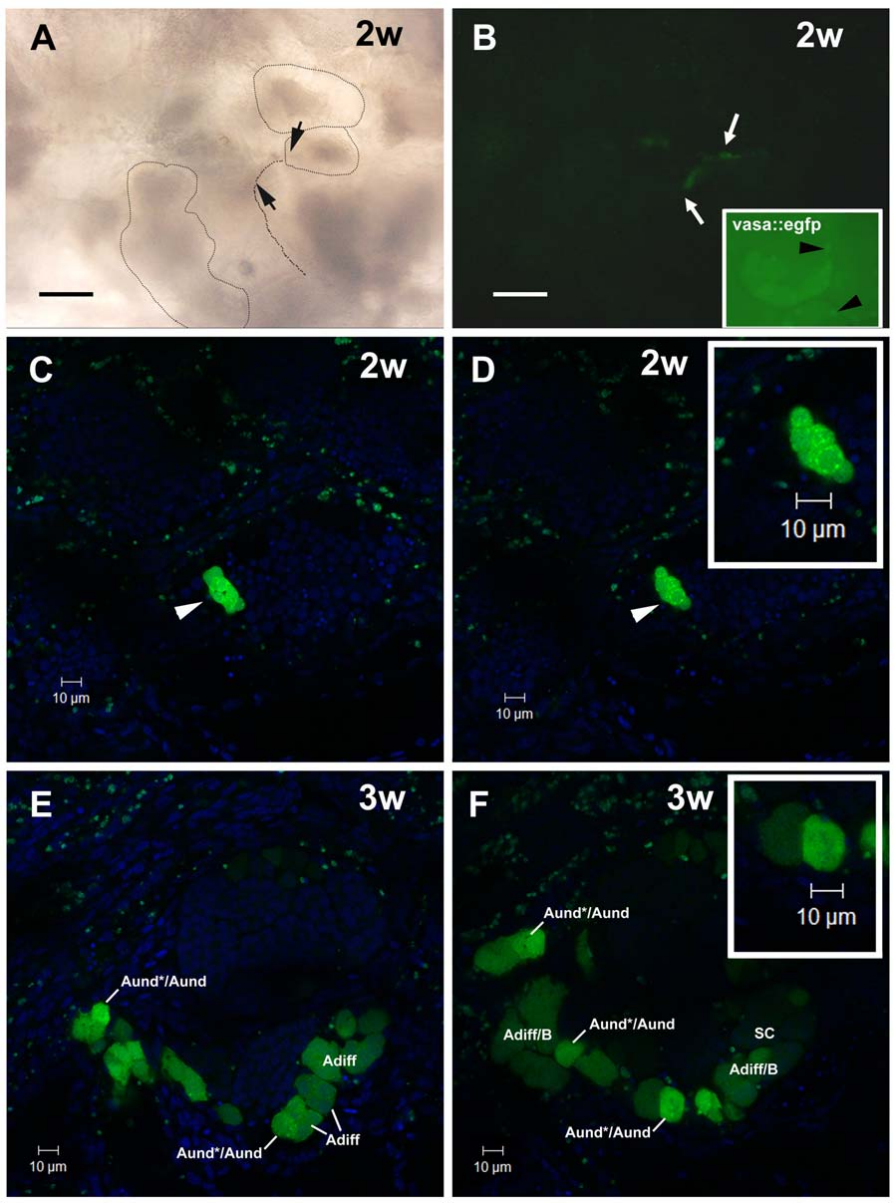
SSC transplantation into male zebrafish recipients. **A,B.** Recipient testes after two weeks (2w) of transplantation analyzed by light (**A**) and fluorescence (**B**) microscopies. Seminiferous tubules are delimited by stippled lines. Arrows indicate the same area in **A** and **B**. Donor cells formed clusters situated near the interstitium in a similar way as observed in *vasa::egfp* testes (see arrowheads in the inset). Nuclei (blue) are stained with DAPI. Scale bars  = 50 µm. **C,D.** CLSM analysis of recipient testes after two weeks (2w) of transplantation. Arrowheads indicate a donor-derived cyst composed of ∼8 cells. **Inset.** High magnification of donor-derived cyst. Nuclei (blue) are stained with DAPI. **E,F.** Recipient testes after 3 weeks (3w) of transplantation analyzed under CLSM. Donor-derived cysts increased their number and size, being found at different stages of spermatogenesis. Type A undifferentiated spermatogonia (A_und*/_A_und_), type A differentiating spermatogonia (A_diff_), type B spermatogonia (B), and spermatocytes (SC). **Inset** shows a high magnification of type A undifferentiated spermatogonia. Nuclei (blue) are stained with DAPI.

**Figure 6:**
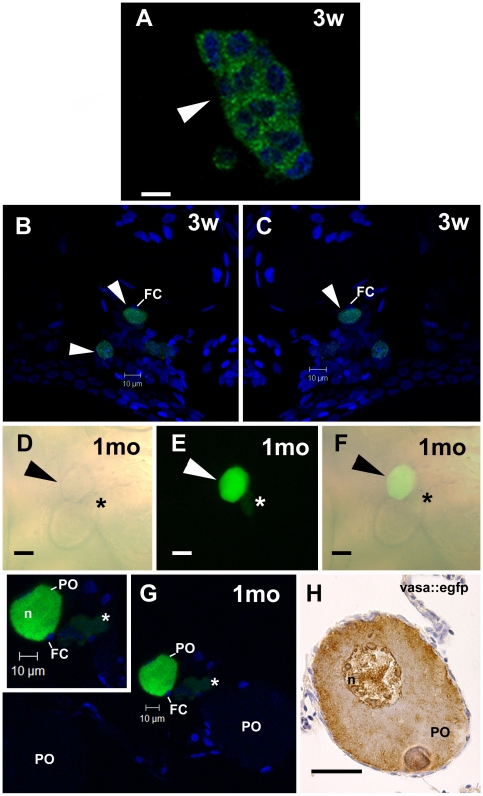
SSC transplantation into female zebrafish recipients analyzed under CLSM. **A.** A GFP cell cluster-derived from transplanted SSC after three weeks (3w) of transplantation. Nuclei (blue) are stained with DAPI. Scale bar  = 10 µm. **B,C.** Arrowhead indicates an early donor-derived oocytes surrounded by follicle cells (FC) after three weeks (3w) of transplantation. Nuclei (blue) are stained with DAPI. **D**–**F.** Arrowhead indicates an advanced GFP oocyte, which was originated from transplanted SSC into zebrafish ovaries after one month (1mo) of transplantation. Note a small GFP cell cluster (asterisk) near the donor-derived oocyte. Light (**D**) and fluorescence (**E**) microscopies, and overlay of both (**F**). Scale bars  = 25 µm. **G.** The same oocyte in **D**–**F** examined under CLSM. A green donor-derived perinucleolar oocyte (PO), endogenous perinucleolar oocyte (PO), small GFP cell cluster (asterisk), and nucleus (n) are shown. **Inset.** High magnification of advanced donor-derived oocyte. Nuclei (blue) are stained with DAPI. **H.** Perinucleolar oocyte from *vasa::egfp* ovaries immunostained for gfp. Compare similar *vasa* expression pattern between donor-derived oocyte and perinucleolar oocyte from *vasa::egfp* ovaries. Scale bar  = 25 µm.

After two weeks of transplantation, donor cells colonized the recipient's seminiferous epithelium, and formed clusters, which were situated near the interstitial compartment (Figure 5A,B). Using CLSM, we found that these clusters were composed of ∼8 cells/cyst (Figure 5C,D; Video S2). Three weeks after transplantation, donor-derived cysts had increased in number and size, and were found at different stages of spermatogenesis (e.g. differentiating type A spermatogonia, type B spermatogonia and spermatocytes) along the recipient's seminiferous epithelium (Figure 5E,F; Video S3). Donor type A undifferentiated spermatogonia (A_und*_ and A_und_) were also found in the recipient seminiferous epithelium (Figure 5E,F). The *vasa* expression pattern in donor-derived germ cells was the same as observed in *vasa::egfp* testes immunostained for GFP, or in wild-type testes immunostained for Vasa (Figure S6). The transplantation efficiency was considered not high (30%) as colonization and donor-derived spermatogenesis was observed in about 3 out of 10 recipients. With regard to SSC transplantation into females, cell clusters derived from transplanted SSC were found in recipient ovaries after three weeks of transplantation (Figure 6A, Video S4). Small GFP-positive oocytes at an early stage of oocyte development were also found in recipient females (Figure 6B,C). Surprisingly, these male-derived germ cells progressed into oocyte development, and gave rise to advanced oocytes after one month of transplantation (Figure 6D–F; Video S5). Clusters of GFP-positive cells, possibly clones of oogonia, were also found near male-derived oocytes (Figure 6G,H). The presence of GFP-positive cells in both transplanted males (three weeks after transplantation) and females (one month after transplantation) was also confirmed by PCR analysis (Figure S7).

### Hormonal characterization and gene expression in busulfan-depleted zebrafish

**Figure 7:**
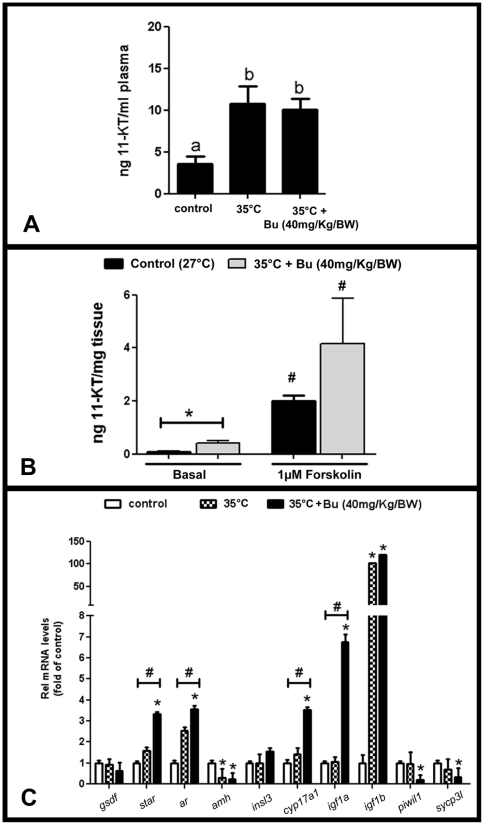
Characterization of male recipients prior SSC transplantation. **A.** 11-KT plasma levels (ng/ml plasma) from zebrafish kept at 27°C (control) (n = 7), 35°C (n = 11), and busulfan-depleted animals (n = 19). Bars represent the mean±SE. Different letters mean significant differences (p<0.05) among groups. **B.** In vitro 11-KT (ng/mg testis) release from control (27°C) (n = 7) and busulfan-depleted testes [35°C + Bu (40 mg/Kg/BW)] (n = 7) in basal and 1 µM forskolin-induced. Bars represent the mean±SE. * means significant differences (p<0.05) between control and depleted in the same experimental condition (Student unpaired t-test). # indicates significantly higher (p<0.05) than the respective basal release (Student paired t-test). **C.** Relative mRNA levels of *gsdf, star, amh, insl3, cyp17a, igf1a, igf1b, piwil1* and *sycp3l* from control (27°C) (n = 7), 35°C (n = 4), and busulfan-depleted testes [35°C + Bu (40 mg/Kg/BW)] (n = 12). Bars represent the mean±SE of relative mRNA levels normalized to *β-actin1* or *ef1α*, and expressed as fold of relative control (27°C) mRNA levels. * and # are significantly different (p<0.05) from control levels (Student unpaired t-test) and among the groups (ANOVA), respectively.

The relatively low efficiency of colonization after transplantation triggered studies on the recipient's testicular microenvironment prior to transplantation. The plasma levels of 11-KT were ∼3 times higher in fish kept at 35°C (with or without busulfan injection) than in non-treated zebrafish kept at 27°C (Figure 7A). The temperature-induced elevation of 11-KT plasma levels was similar to the stimulatory effect of hCG (10 IU/g) or recombinant zebrafish Fsh (100 ng/g) [33]. Also, testicular 11-KT release in primary culture was higher from tissue of males exposed to 35°C and busulfan than from testis tissue of control males kept at 27°C, in particular with regard to basal release (7-fold higher), while the difference in forskolin-stimulated androgen did not reach statistical significance (Figure 7B). Quantifying the expression of selected genes revealed that transcript levels of steroidogenesis and androgen signaling genes (*star, ar, cyp17a1*) had increased 3-fold in busulfan-depleted testes (Figure 7C). In most cases, exposure to 35°C alone did not change gene expression. There are two interesting exceptions. The transcript levels of *amh* were significantly down-regulated at 35°C and in busulfan-depleted testes, while *igf1b* transcript levels were strongly up-regulated. Expression of germ cell-specific genes, such as, *piwil1* and *sycp3l* significantly decreased only on busulfan-treated animals (Figure 7C), while *gsdf* and *insl3* mRNA levels did not change among the groups (Figure 7C).

## Discussion

Using a BrdU pulse-chase approach, we identified the LRC population in zebrafish testis. Within this category of testicular cells, somatic cells were found (e.g. Sertoli and interstitial cells), but type A undifferentiated spermatogonia (A_und*_ and A_und_) were the only germ cells able to retain the label after 18 and 25 days of chase, constituting therefore the putative SSCs. Two distinct patterns of BrdU dilution were found among the two subtypes of type A spermatogonia in zebrafish. The BrdU dilution kinetics for type A_und_ showed a rapid decline that stabilized at the 18^th^ day after chase, while the percentage of BrdU-positive A_und*_ spermatogonia remained constant. Applying mathematical modeling analysis to hematopoietic stem cells (HSCs) [39], a similar biphasic profile of BrdU dilution was described. The initial, rapid decline was attributed to the faster turnover of activated cells, while the subsequent, decelerated decline resulted from the slow turnover of quiescent cells. This suggested the presence of a heterogeneous population of HSCs which can be activated or deactivated according to the systemic needs [39]. Likewise, the biphasic profile found in zebrafish testes might be attributed to functional differences between the two subtypes of spermatogonia, in which A_und_ would be the more rapidly dividing, “active” population (related to differentiation and rapid proliferation, as indicated by the more rapid loss of BrdU), and A_und*_, the slow-cycling, “reserve” population (related to slow self-renewal proliferation, as indicated by the relatively stable BrdU labeling index). Also in the human testis, two types of single A spermatogonia (pale and dark) are present, and might play distinct roles as “reserve” versus “active” stem cell, respectively [40], [41]. In another teleost fish, in female medaka (*Oryzias latipes*), a BrdU pulse-chase experiment also revealed two distinct populations of oogonial stem cell, one being fast and the other a slow-dividing cell population [42]. Across the vertebrates, it seems that tissues with constant demand for stem cells to proliferate and generate differentiated cells, two populations of stem cells are required; one to rapidly expand and produce differentiated cells, and another which rarely divides and functions as “reserve” in case of insults.

According to the “immortal strand hypothesis” [43], stem cells divide asymmetrically, since a hypothetical mechanism would sort chromatids containing the original template DNA strands in one daughter cell, passing therefore the new strands to the other daughter cell [44]. Hence, in a stem cell self-renewal division, the new stem cell would inherit the original templates, the other daughter cell, destined to differentiate, would inherit the new strands. Recently, it has been demonstrated that the “immortal strand hypothesis” is not exclusive of self-renewing cells, but also of divisions in which daughter cells adopt divergent fates [45]. In this case, daughter cells inheriting the original templates would also retain the more immature phenotype, whereas daughter cells inheriting the newer templates acquired a more differentiated phenotype [45]. The BrdU dilution for A_und_ suggests the existence of two types of template DNA strand segregation, one random type, possibly reflecting the rapid loss of the label up to the 18th day of chase, and one non-random type (asymmetrical division), which could explain the retention of BrdU after 18 and 25 days of chase. We speculate that the asymmetrical division of A_und_ might prevent its exhaustion during spermatogenesis, maintaining, therefore, a subset of immature A_und_. An asymmetrical division of type A undifferentiated spermatogonia was suggested previously in zebrafish based on morphological evidence [2].

The LRC assay also revealed the precise location of stem cells, and consequently the characterization of the neighboring cells that form the stem cell niche [46]–[48]. As in rodents [6], [7], we found that slow-cycling BrdU-labeled type A undifferentiated spermatogonia (A_und*_ and A_und_) were preferentially situated in regions of the seminiferous tubules adjacent to the interstitial compartment. This observation suggests that elements of the SSC niche are probably conserved across vertebrates. The preferential location of SSC close to the interstitial compartment in rodents may be related to a locally high concentration of androgens which inhibit spermatogonial differentiation [49]. 11-KT is the main androgen in zebrafish [37] and other teleost fish, and is able to support full spermatogenesis in testis tissue culture of juvenile Japanese eel (*Anguilla japonica*) [50] or adult zebrafish [38]. Moreover, induction of androgen insufficiency in adult male zebrafish inhibited the differentiation of type A to type B spermatogonia [51]. Although the possible role(s) of androgen signaling in regulating zebrafish SSC activity still have to be clarified, the available evidence in fish strongly suggests that, different from mammals, androgens stimulate spermatogonial differentiation [2]. On the other hand, estrogens stimulated SSC self-renewal in Japanese eel [52], so that Leydig cells may be relevant as a source for aromatizeable androgens, thereby contributing directly and/or indirectly to the zebrafish SSC niche. Moreover, Leydig cell paracrine signaling might influence SSC behavior, since colony stimulating factor 1 (Csf1) produced by Leydig and myoid cells is an extrinsic stimulator of SSC self-renewal in mice [10].

Endothelial cells support the expansion of normal and malignant stem cells not only by delivering oxygen and nutrients, but also by the paracrine release of endothelial cell growth factors and trophogens, which are referred to as “*angiocrine factors*” [53]. Thus, endothelial cells have been pointed out as an important element of the stem cell niche in several systems, including the rodent SSC niche [8], [54]. In the zebrafish model, the distribution of SSC candidates (A_und*_ and A_und_) close to blood vessels might indicate an involvement of endothelial cells in regulating SSC function. While there is no information on angiocrine signaling in the zebrafish testis, it has been shown that platelet-derived endothelial cell growth factor (PD-ECGF) induced SSC self-renewal in Japanese eel [55], [56].

The functional capacity of the identified SSC candidates in zebrafish was investigated by transplantation assays. In this assay, SSC candidates were transplanted into zebrafish in which endogenous spermatogenesis had been depleted by busulfan, similar to the technique developed for rodents and others species [14]–[17], [19]. It seems that similar to tilapia [16], zebrafish spermatogenesis is more sensitive to busulfan at elevated temperatures (35°C), resulting in 88% of spermatogenic tubules showing Sertoli cells only after 10 days of treatment. Interestingly, spermatogenesis recovered quickly after having passed the nadir of busulfan-induced depletion. It has been shown previously that proliferation of surviving, undifferentiated spermatogonia is greatly enhanced when the number of differentiating spermatogonia is reduced following busulfan exposure [57], [58]. In addition, differentiated spermatogonia can act as “potential stem cells”, shifting their nature of transit-amplifying cells to self-renewal in order to rapidly recover spermatogenesis in depleted testes [59]. Analyzing the composition of type A spermatogonia in normal versus busulfan-depleted testes in zebrafish, we have demonstrated a relative increase in the population of A_und_, and a relative decrease in the population of A_und*_ spermatogonia in busulfan-depleted testes. Then, considering A_und_ as the “active” stem cell, our results suggest that the spermatogenic recovery after busulfan treatment might be associated with a preponderance of the active stem cell population A_und_.

The treatment with busulfan induced an increase in androgen plasma levels as well as an increase in testicular androgen release. This might reflect a suppression of the negative feedback on gonadotropin release. In rat, the negative feedback on FSH release exerted by germ cells via Sertoli cell-derived inhibin is transiently eliminated after busulfan treatment [60], explaining the increase in circulating FSH levels between 6 and 10 weeks after busulfan injection [61], [62]. In mammals, androgen plasma levels were not elevated in busulfan-treated animals [60]–[62]. However, in fish, also Leydig cells express the receptor for Fsh, which is a strongly steroidogenic gonadotropin in fish (e.g. [33], [63], [64]). Hence, we attribute the increased androgen levels after busulfan treatment to higher Fsh plasma levels. Consistent with the elevated androgen production, busulfan treatment increased the mRNA levels of transcripts encoding proteins involved in steroidogenesis, such as *star* and *cyp17a1*. The down-regulation of *amh* mRNA expression in busulfan-depleted testes also seems coherent with higher androgen levels, since androgens down-regulated *amh* expression in juvenile Japanese eel testis and stimulated spermatogonial differentiation towards meiosis [65]. Moreover, since Sertoli cells also proliferate in the adult fish testis, in particular those associated with expanding spermatogonial cysts [23], [24], the busulfan treatment may have depleted the proliferating Sertoli cell population as well, thereby contributing to lower levels of *amh* mRNA, and possibly also to the arrest in spermatogenesis. With regard to the expression of *igf* gene family members, a strong up-regulation in particular of *igf1b* mRNA levels has been observed. This is interesting in the context of ongoing work showing that recombinant zebrafish Fsh increases *igf1b* levels in adult testis tissue culture (unpublished data). Spermatogonial proliferation was stimulated in newt testis in primary tissue culture by exposure to Igf1 [66]. Since Sertoli cells also express a factor (similar to platelet-derived endothelial cell growth factor) that stimulates SSC self-renewal in Japanese eel [55], busulfan-induced changes in the number and/or physiological state of Sertoli cells might have resulted in a more generalized imbalance of factors released by Sertoli cells and relevant for SSC self-renewal and differentiation. Taken together, our data suggest that busulfan treatment might create a microenvironment in zebrafish testes where the balance of somatic (mainly Sertoli) cell-derived factors has shifted to a reduced level of factors supporting self-renewal and/or an elevated level of factors stimulating differentiation. While this might facilitate the fast recovery after the severe loss of germ cells induced by busulfan, it may also influence the behavior of transplanted cells. Thus, transplanted SSCs may differentiate rather than self-renew, possibly limiting the efficiency of donor-colonization (30%) in the recipient seminiferous tubules; however, a relatively low efficiency of donor-derived colonization is not exceptional for zebrafish and has been observed in other vertebrates as well [67]. Future studies will aim at weakening the pro-differentiation or strengthening the self-renewal environment, for example by an estrogen treatment [51] of the recipients.

Despite of the limited efficiency, we have taken important steps towards the development and standardization of SSC transplantation assay to confirm the “stemness” of SSC candidates in zebrafish. Transplantation of a FACS-enriched fraction of A_und*_ and A_und_ into busulfan-depleted testes showed that stem cell candidates were able to colonize, self-renew, and to differentiate in the recipient seminiferous tubules. This confirms the presence of stem cells in the transplanted pool of type A undifferentiated spermatogonia. Moreover, spermatogonial colonization started from regions within the recipient's seminiferous tubules which are adjacent to the interstitial compartment, the putative SSC niche in zebrafish testes. This might indicate that the SSC niche creates a cellular and molecular environment which is optimal for colonization and development of transplanted SSCs. Since zebrafish have been used as a model for understanding the mechanisms of testicular germ cell tumors [68], and most of these tumors have an unknown etiology, a standard experimental design including reciprocal transplantation of germ cells from affected donors to wild-type testes and vice versa could be applied to evaluate whether the defect is on Sertoli cells or intrinsic to the germ cells. Another important application of germ cell transplantation is transgenesis through the male germ line using transplantation of transfected germ cells [3], [19]. This might be an option to create transgenic animals in a shorter time than is possible with conventional methods [19].

Finally, we have demonstrated the plasticity of the transplanted spermatogonia by placing the SSC candidates into a different microenvironment, i.e. into an ovary. SSC candidates were able to colonize recipient ovaries and differentiate into female germ line cells.

We present in the current work characteristics of zebrafish SSC candidates (A_und*_ and A_und_), their niche, and their functional capability to both self-renew and differentiate in recipient testes, as well as their remarkable plasticity in recipient ovaries. To our knowledge, this is the first work to report on a SSC niche in fish. As in rodents, SSC location in zebrafish was characterized within areas of the seminiferous tubules which are opposite to the interstitial compartment, suggesting the influence of interstitial elements on SSC self-renewal and maintenance. We developed and standardized SSC transplantation techniques in male and female zebrafish, showing donor-derived spermatogenesis and male-derived oocytes, respectively, after transplantation. Thus, we introduced SSC transplantation in zebrafish as a promising technique for the study of SSCs.

## Supporting Information

Figure S1 Effects of different temperatures on zebrafish spermatogenesis. A. More advanced labeled germ cell after 2 h, 12 h, 1d, 2d, 3d, 4d, 5d, 6d of 3H-thymidine injection in zebrafish males kept at 20°C, 27°C, 30°C, and 35°C. Leptotene/zygotene (L/Z) and pachytene (P) spermatocytes, metaphase I (MI), initial spermatids (E1), intermediate spermatids (E2), final spermatids (E3) and spermatozoa (Z). Scale bars  = 10 µm. Cells were considered labeled when four to five or more grains were present over the nucleus in the presence of low-to-moderate background (i.e., very few grains per histological field observed under oil immersion). Black squares indicate the time in which labeled spermatozoa were found in the lumen of zebrafish testes at different temperatures. B. Histogram showing the combined duration of meiotic and spermiogenic phases at different temperatures. Spermatogenesis did not progress beyond the first meiotic division at 35°C. X axis represents the time, whereas y axis the 3H-thymidine labeled germ cell [Leptotene/zygotene (L/Z), pachytene (P) and diplotene (D) spermatocytes, metaphase I (MI), initial spermatids (E1), intermediate spermatids (E2), final spermatids (E3) and spermatozoa (Z)]. C–E. Histological sections of zebrafish testes at 27°C, 30°C, and 35°C. Sperm free and a massive germ cell apoptosis is seen in zebrafish seminiferous tubules at 35°C. Scale bars  = 50 µm.
(4.05 MB TIF)

Figure S2 Standardization of SSC transplantation techniques. A. Transplantation device, in which a glass capillary needle is coupled to a peristaltic pump. B,C. Standardization of transplantation via using trypan blue to monitor the efficiency of the injections. Note trypan blue inside the testis (B) and ovaries (C). D,E. Genital pore histological sections stained with toluidine blue (male - D, female - E). Genital pore measurements: 120×80 µm dimensions, 63° angle (female); 75×50 µm dimensions, 60° angle (male). Anus (A), genital pore (G, arrow), and urethra (U). Scale bars  = 100 µm. F. Ovary cell transplantation throughout injections into the lateral body wall (indicated by stippled line). G. Trypan blue was used to monitor the specificity of the injection. Note trypan blue inside the ovaries.
(2.98 MB TIF)

Figure S3 Scatter plot to check the stability of 18S rRNA, β-actin1 and ef1α mRNAs as a housekeeping gene in 35°C (n = 4), and depleted testes (35°C + busulfan 40 mg/Kg/BW) (n = 12). Each dot in the scatter plot represents the average Cq-value of duplicate measurements for each fish in the different experimental condition. Stability was seen only between β-actin1 and ef1α. Control (27°C) was not shown, but followed the same pattern.
(0.26 MB TIF)

Figure S4 The hypothetical SSC niche in zebrafish testes. SSC niche is indicated by a red circled line. The niche is constituted by elements of the tubular and interstitial compartments (IC) such as: Sertoli cells (SE); basement membrane (BM); peritubular myoid cells (PM); Leydig cells (LE), blood vessels (BV); and other interstitial elements. Type A undifferentiated spermatogonia (Aund*/Aund), type A differentiated spermatogonia (Adiff), type B early spermatogonia (B early) and type B late spermatogonia (B late) and spermatozoa (SZ) are illustrated.
(2.57 MB TIF)

Figure S5 Preparation of female zebrafish recipients for SSC transplantation. A–C. Ovaries histological sections of ovoposition-induced females stained with PAS (Periodic acid Schiff). Arrows indicate postovulatory follicles after ovoposition. Note in C the available follicle cells which can support the transplanted SSC development. Scales bars  = 250 µm (A), 100 µm (B), 25 µm (C). D. Number of postovulatory follicles (POFs)/mm2 of ovary in control (n = 5) and ovopositioned females (n = 5). Bars represent the mean± SE. * means significant differences (p<0.05) between the groups (Student unpaired t-test).
(1.53 MB TIF)

Figure S6 Vasa expression pattern during male germ cell development using gfp immunodetection in sections of vasa::egfp testes (A), or vasa immunodetection in cryosections of wild-type testes (B). Type A undifferentiated spermatogonia (Aund*/Aund), type A differentiating spermatogonia (Adiff), type B spermatogonia (B), spermatocytes (SC), spermatids (ST), spermatozoa (SZ). Scale bars  = 25 µm (A) and 10 µm (B).
(2.83 MB TIF)

Figure S7 PCR analysis for GFP detection using primers located in the YFP gene, 2% agarose gel containing ethidium bromide, showing the detection of a faint background band. A. vasa::egfp testes were used as a positive control. Transplanted male after 3 weeks of transplantation, transplanted female after 1 month of transplantation, H2O water as negative control. Bands at left side are DNA markers from SMART ladder (Eurogentec). B. PCR detection using primers located in the PD-ECGF (plated-derived endothelial cell growth factor) gene as positive control for genomic DNA in the different individuals, 2% agarose gel containing ethidium bromide, showing the detection of a faint background band. Bands at left side are DNA markers from SMART ladder.
(0.82 MB TIF)

Video S1 Z stack from vasa::egfp testis showing the distribution of vasa positive cells in the zebrafish seminiferous tubules.
(1.63 MB WMV)

Video S2 Z stack from wild-type zebrafish testis 2 weeks after SSC transplantation. Note a donor-derived cyst composed of ∼8 cells in the seminiferous epithelium.
(0.83 MB WMV)

Video S3 Z stack from wild-type zebrafish testis 3 weeks after SSC transplantation. SSCs were able to colonize recipient testis and differentiate into daughter cells committed with the spermatogenic process. Donor-derived cysts at different stages of zebrafish spermatogenesis were found in recipient seminiferous epithelium.
(0.81 MB WMV)

Video S4 Z stack from ovaries fragments 3 weeks after SSC transplantation into female zebrafish ovaries. Small cell clusters-derived from SSC were seen in female ovaries.
(1.10 MB WMV)

Video S5 Z stack from ovaries fragments 1 month after SSC transplantation. SSCs were able to colonize female ovaries and differentiate into female germ cell line. Note a male-derived oocyte in the female recipient ovary.
(0.60 MB WMV)
