# Supplementary figures and images for: Spermatogonial Stem Cell Niche and Spermatogonial Stem Cell Transplantation in Zebrafish
Source: PLoS One. 2010 Sep 20;5(9):e12808. doi: 10.1371/journal.pone.0012808 (PMC2942835; doi:10.1371/journal.pone.0012808)

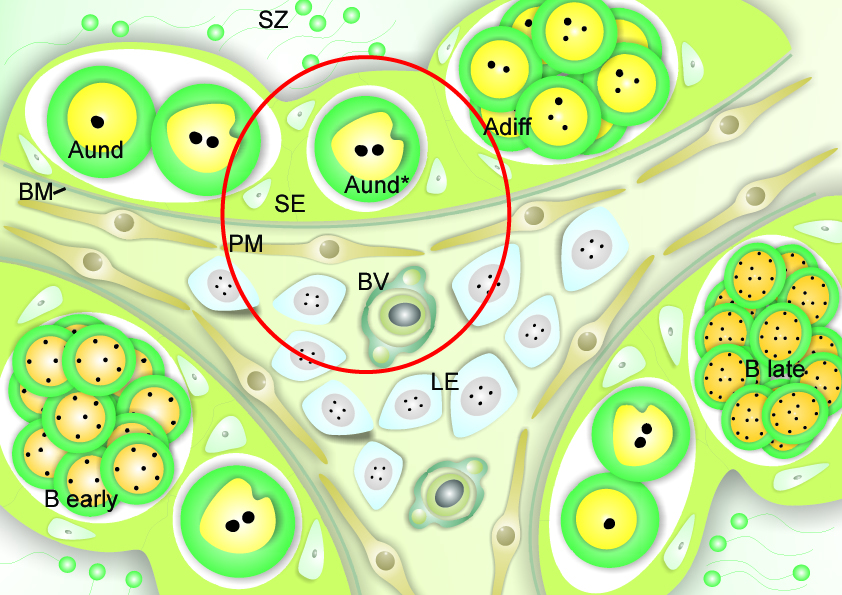

Supplement: Figure S4 — The hypothetical SSC niche in zebrafish testes. SSC niche is indicated by a red circled line. The niche is constituted by elements of the tubular and interstitial compartments (IC) such as: Sertoli cells (SE); basement membrane (BM); peritubular myoid cells (PM); Leydig cells (LE), blood vessels (BV); and other interstitial elements. Type A undifferentiated spermatogonia (Aund*/Aund), type A differentiated spermatogonia (Adiff), type B early spermatogonia (B early) and type B late spermatogonia (B late) and spermatozoa (SZ) are illustrated. (2.57 MB TIF) [file pone.0012808.s004.tif]

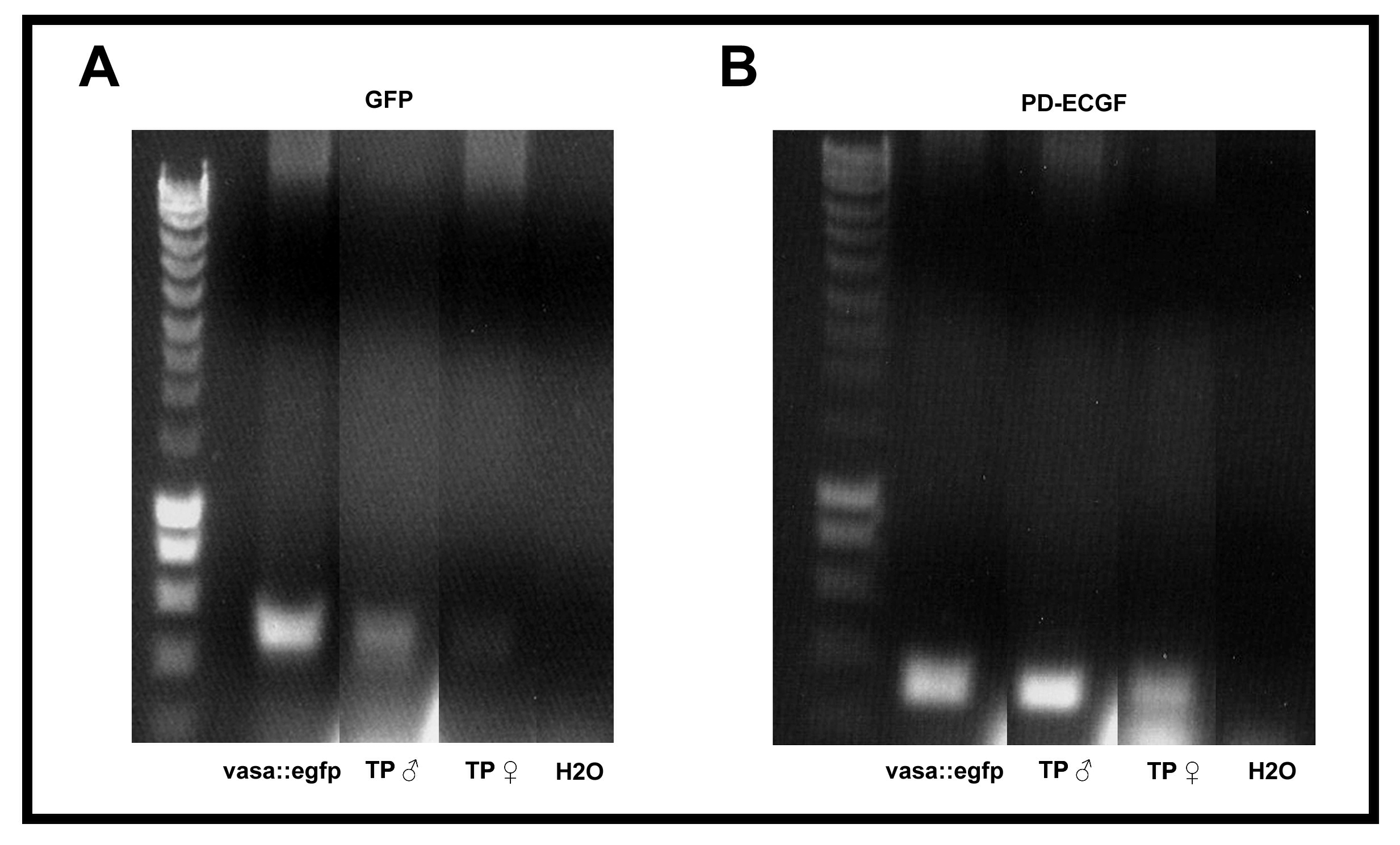

Supplement: Figure S7 — PCR analysis for GFP detection using primers located in the YFP gene, 2% agarose gel containing ethidium bromide, showing the detection of a faint background band. A. vasa::egfp testes were used as a positive control. Transplanted male after 3 weeks of transplantation, transplanted female after 1 month of transplantation, H2O water as negative control. Bands at left side are DNA markers from SMART ladder (Eurogentec). B. PCR detection using primers located in the PD-ECGF (plated-derived endothelial cell growth factor) gene as positive control for genomic DNA in the different individuals, 2% agarose gel containing ethidium bromide, showing the detection of a faint background band. Bands at left side are DNA markers from SMART ladder. (0.82 MB TIF) [file pone.0012808.s007.tif]
